# Information Extraction from the Text Data on Traditional Chinese Medicine: A Review on Tasks, Challenges, and Methods from 2010 to 2021

**DOI:** 10.1155/2022/1679589

**Published:** 2022-05-13

**Authors:** Tingting Zhang, Zonghai Huang, Yaqiang Wang, Chuanbiao Wen, Yangzhi Peng, Ying Ye

**Affiliations:** ^1^Chengdu University of Traditional Chinese Medicine, Chengdu, China; ^2^Chengdu University of Information Technology, Chengdu, China

## Abstract

**Background:**

The practice of traditional Chinese medicine (TCM) began several thousand years ago, and the knowledge of practitioners is recorded in paper and electronic versions of case notes, manuscripts, and books in multiple languages. Developing a method of information extraction (IE) from these sources to generate a cohesive data set would be a great contribution to the medical field. The goal of this study was to perform a systematic review of the status of IE from TCM sources over the last 10 years.

**Methods:**

We conducted a search of four literature databases for articles published from 2010 to 2021 that focused on the use of natural language processing (NLP) methods to extract information from unstructured TCM text data. Two reviewers and one adjudicator contributed to article search, article selection, data extraction, and synthesis processes.

**Results:**

We retrieved 1234 records, 49 of which met our inclusion criteria. We used the articles to (i) assess the key tasks of IE in the TCM domain, (ii) summarize the challenges to extracting information from TCM text data, and (iii) identify effective frameworks, models, and key findings of TCM IE through classification.

**Conclusions:**

Our analysis showed that IE from TCM text data has improved over the past decade. However, the extraction of TCM text still faces some challenges involving the lack of gold standard corpora, nonstandardized expressions, and multiple types of relations. In the future, IE work should be promoted by extracting more existing entities and relations, constructing gold standard data sets, and exploring IE methods based on a small amount of labeled data. Furthermore, fine-grained and interpretable IE technologies are necessary for further exploration.

## 1. Introduction

Traditional Chinese medicine (TCM), a unique theoretical and practical approach to the treatment of diseases, has played an indispensable role in the health care of the Chinese population for several thousand years [1]. According to the latest global medical outline (Ver.2019) of the World Health Organization (WHO), TCM is a popular and effective complementary and alternative medicine in preventing and treating many diseases [2]. An example is the integrated use of TCM in the treatment of COVID-19, which was shown to be more effective than the use of modern therapies alone [3, 4].

Textual data is the most common data type in TCM. Throughout history, the rich data source of TCM that dates back thousands of years is scattered in different text formats in paper versions such as case notes, manuscripts, printed books, and publications [5]. All these text data contain valuable medical information such as manifestations of disease, diagnostic rules, therapeutic methods, and effective prescriptions. Hence, it is a crucial carrier of TCM knowledge and contributes to the inheritance and dissemination of TCM. However, human labor is insufficient to deal with the increasing accumulation of text data, and hence automated TCM knowledge discovery is necessary and urgently needed.

With the continuous development of information technology and artificial intelligence (AI), data mining is an approach that can be used to obtain information from many sources and is increasingly applied in mining of medical texts, including the TCM literature [6]. More fortunately, informatization of TCM text sources has brought opportunities for the deep utilization of diverse TCM texts in recent decades [7]. However, the majority of electronic TCM texts are stored as unstructured data, including a tremendous amount of case notes, with chief complaint and history of present illness, abstracts of publications, Web data, etc., that cannot be directly used for text mining. Therefore, the imperative task before data mining can be conducted is to transform TCM narratives into structured data.

**Figure 1:**
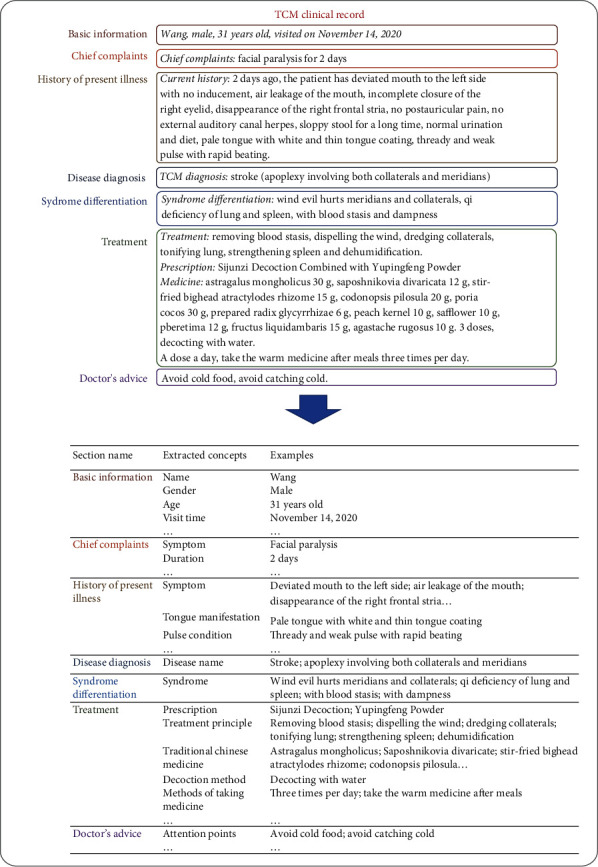
Example of extracting information from a TCM clinical record.

Information extraction (IE) techniques, an important part of natural language processing (NLP), have assisted automatic information acquisition from TCM texts and have been increasingly used in text mining in recent years [8, 9]. By building large-scale and comprehensive structured data sets, TCM texts can be widely used in clinical applications such as information retrieval [10], computer-aided syndrome differentiation and diagnosis [11, 12], and analysis of prognosis [13]. An example of extracting information from a clinical record of TCM is shown in Figure 1.

To our knowledge, there are many IE tools that can extract information from English language unstructured data, such as MedLEE [14], MetaMap [15], and MedTagger [16]. However, owing to the differences between the Chinese and Western languages, such as different word segmentation methods, different syntactic structures, and word and sentence ambiguity, these tools or systems of IE in English cannot be transplanted directly to Chinese IE tasks, and TCM texts are even more challenging. With this background, more approaches were explored to extract information from different types of TCM text data, and IE from TCM texts has shown encouraging improvements accordingly [17, 18]. Although previous research has summarized some IE work in TCM [19–21], the new advanced technologies and emerging methods need to be further summarized and synthesized, for example, improved deep learning approaches and more types of extracted information [22, 23]. In this study, we searched four literature databases for articles published from 2010 to 2021 that focused on the use of NLP methods to extract information from unstructured TCM text data. Based on what we found in the articles, we assessed the status of IE from TCM sources as related to (1) data sources and extracted information, (2) challenges, (3) NLP frameworks and models, and (4) key findings. We conclude with some recommendations for future studies.

## 2. Methods

Review procedures were carried out under the guidance of Preferred Reporting Items for Systematic Reviews and Meta-Analyses (PRISMA) [24], which lists priority report items for systematic reviews and meta-analyses. Under the instructions in PRISMA, we undertook our review in three steps: (1) article search, (2) article selection, and (3) data extraction and synthesis. The PRISMA checklist is attached as Supplementary 1.

### 2.1. Article Search

**Figure 2:**
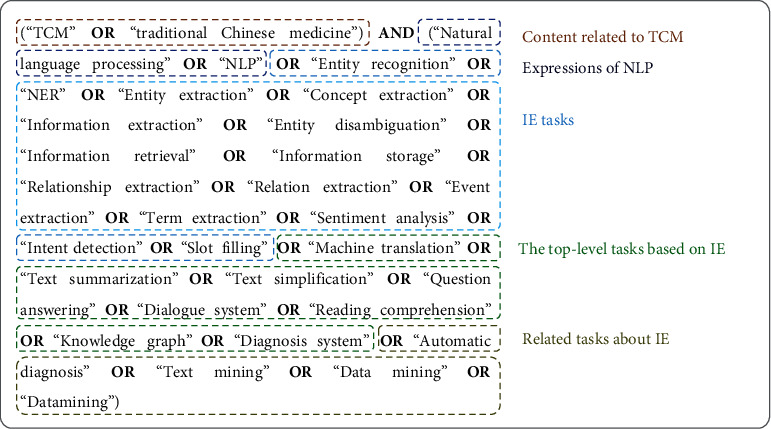
The search strategy and reasons for each search string category.

We searched PubMed, Web of Science, EMBASE, and Engineering Village on 31 December 2021 to identify all potentially relevant publications related to extracting information from TCM text data. The search strategy and the reasons for each search string category are shown in Figure 2. The selected keywords and the associations between these keywords were identical in the searches for each database. Queries were limited to English language. The time period ranged from 2010 to 2021. The searches returned 564 articles from PubMed, 435 from Web of Science, 427 from Embase, and 779 from Engineering Village. Subsequently, the titles, abstracts, keywords, and other information from all retrieved papers were imported into EndNote X7 reference management software for data selection. We made use of the “find duplicates” function to find the 970 duplicated papers. A total of 1,234 publications were retrieved in this step.

### 2.2. Selection of Articles

**Figure 3:**
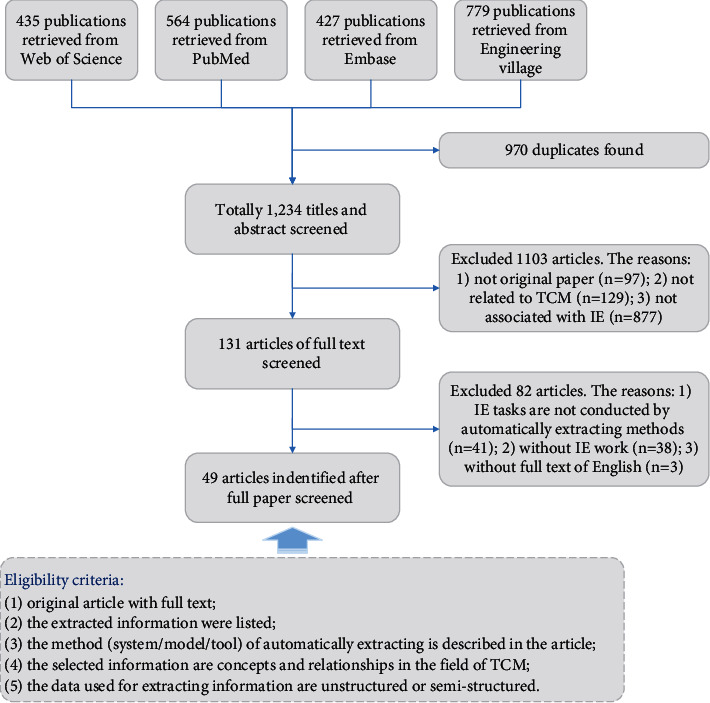
Flow diagram of included articles and eligibility criteria.

We used EndNote X7 reference management software to manage many of the screening and coding processes of our systematic review procedures. Attention was paid to the methodology and content in the selection process. The filtering criteria and eligibility criteria are listed in Figure 3. In the data selection step, the articles were labeled with “include”, “exclude”, and “undetermined” according to the criterion applied. Two reviewers (Zhang, Ye) independently reviewed the titles, abstracts, and full texts of the retrieved publications and an adjudicator (Wang) reviewed the articles with disagreement. One of the two reviewers has background knowledge of TCM, and the other reviewer and the adjudicator are mainly engaged in NLP research. The cooperation of three reviewers with TCM and computer knowledge background ensured the reliability of the paper screening results. For articles on which the adjudicator could not make a decision, the three reviewers discussed together to decide whether to include or exclude the article. After the first screening, 131 articles that were probably related to IE for TCM were included. Afterward, we downloaded the full-text PDF file to conduct the next round of screening. During this step, the methodology and data source for each publication were reviewed to make a final decision. The same selection procedure was used as before: two reviewers screened the full text for all records, and the third adjudicator reviewed the disagreements between them. Finally, a total of 49 eligible papers were determined to meet the review criteria.

### 2.3. Data Extraction and Synthesis

Data extraction from the 49 articles included in the paper selection step was performed manually by two reviews and one adjudicator. First, two reviewers (Zhang and Ye) independently reviewed the articles and extracted information to form an Excel table. The information or items extracted included author, title, year published, data type, data number and source, disease, approach (system/model/tool, method type), extracted information, evaluation measures and performance, comparative evaluations, challenges, objective, and relevant outcomes. After that, an adjudicator (Wang) reviewed the table of papers with disagreements and made a final data sheet. The final table of the extracted data is attached as Supplementary 2. In this paper, our aim is to describe and assess the following aspects of the studies: (1) data sources and extracted information, (2) challenges, (3) NLP frameworks and models, and (4) key findings. On the basis of these results, we conclude with some recommendations for future studies.

## 3. Results of TCM Information Extraction in the Last Ten Years

**Figure 4:**
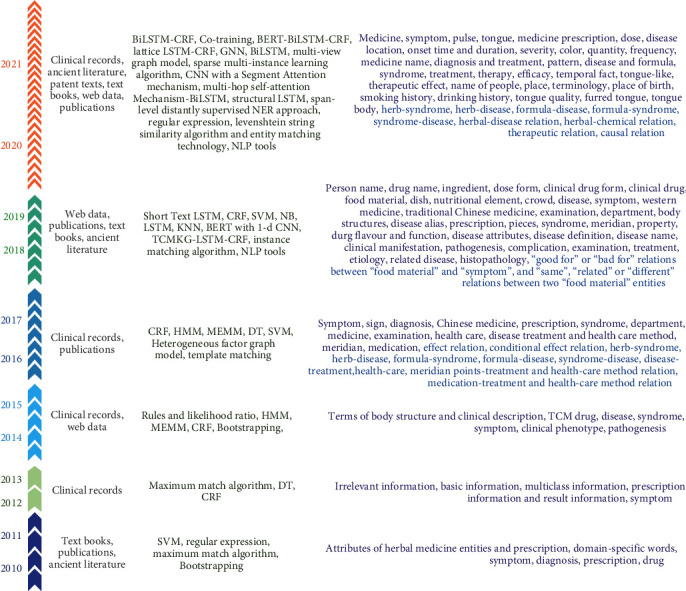
Timeline of data sources, models (or algorithms), and extracted targets of TCM.

We synthesized the data sources, models (or algorithms), and extracted targets of TCM into a timeline, as shown in Figure 4. In Figure 4, the number of colored shapes on the left side represents the number of articles published in the corresponding time interval. Obviously, the number of published papers on IE from TCM is increasing. In particular, from 2020 we counted a total of 25 published papers. The trend of growth in IE work indicates that IE from TCM texts has attracted much attention and is developing rapidly. In terms of IE methods, researchers have explored many deep learning methods in the last two years.

### 3.1. The Tasks of Extracting Information from TCM Texts

#### 3.1.1. Data Sources

**Figure 5:**
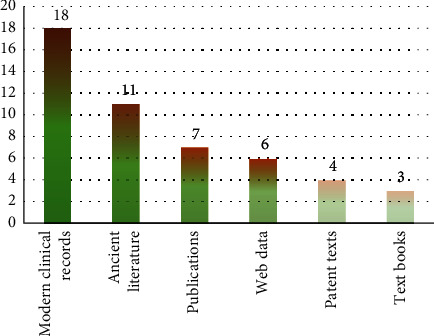
The number of papers enrolled for each type of data source.

The data used and structured in the 49 studies in this review came from six types of TCM free text. The distribution of the number of papers for six types of data source is shown in Figure 5. As shown in Figure 5, clinical records were the most commonly used data source for IE in the TCM domain, followed by the ancient literature and four other types of data source. Given that TCM data varied widely in different data sources, we organized the basic data composition of each data source below, hoping that this would be helpful for future IE research.

*Modern Clinical Records*. Modern clinical records can be divided into resident admission notes (RANs) and outpatient records. RANs, also called inpatient clinical records, are an important source of data for IE tasks and clinical applications. The RANs contain comprehensive information on the patient's hospitalization period, such as basic information, chief complaints, personal history, current history, past history, physical examinations, biochemical examinations, diagnoses, and treatments. In the above sections, the types of TCM data were name, gender, temporal words, symptoms, pulse conditions, tongue manifestations, disease names, syndromes, prescriptions, acupoints, differential symptoms, viscera, etc. Unlike RANs, outpatient records are simple records of the patient's condition at the time of their visit, often in electronic or handwritten (or a transcript of a manuscript) form. Outpatient records are always concise, and only the key information is recorded, such as important symptoms and signs, differential symptoms, diagnoses, and treatments (mainly prescriptions and Chinese medicine). In particular, most of the case records of famous past TCM experts were recorded in the form of outpatient medical records. For example, the record “dizziness for three days” is extracted as chief complaints. In this record, “dizziness” is symptom, and “three days” is duration.

*Ancient Literature*. The ancient literature (also called ancient clinical record) documented by ancient TCM practitioners was always written in an obscure and concise style, without a standard format. Consequently, ancient literature is more unintelligible than modern records. In this review, all ancient literature came from ancient Chinese medicine books, including case records books (case records books record the typical examples of doctors' clinical diagnosis and treatment), comprehensive prescription books (comprehensive prescription books, with diseases and prescriptions as the core, are a summary description of ancient Chinese medicine experts in the process of diagnosis and treatment, not just for a patient), classic books of TCM (the classic books of TCM refer to the four classic works of TCM, including “黄帝内经” (Inner Canon of Yellow Emperor), “难经” (Classic of Questioning), “伤寒杂病论” (Treatise on Cold Pathogenic and Miscellaneous Diseases), and “神农本草经” (Sheng Nong's Herbal Classic)), etc. There were a number of significant data types in the ancient literature, including disease names, symptoms, syndromes, zang-fu organs, yin and yang, the five elements, meridians and collaterals, acupuncture points, prescriptions, medicine, explanations for the mechanism of TCM, etc. For example, in an ancient record “妇人怀孕, 腹中疼痛, 当归芍药散主之” (pregnant women has abdominal pain, angelica paeonia powder can treat it), “pregnant women has abdominal pain” is symptom, and “angelica paeonia powder” is prescription name.

*Journal Publications*. There was much useful data in countless published papers, such as titles, abstract, keywords, text sections, and references. Taking the “abstract” as an example, the abstract is easily accessible and contains a wealth of information, such as objectives, methods, results, and conclusions.

*Text Books*. Text books used as the IE sources include text books compiled by modern experts and books based on the original text of ancient books, such as *TCM diagnostics* and *TCM basic theory*. The data composition of the text books was similar to that of ancient literature and clinical records. Additionally, there were many explanations and descriptions of TCM theories in text books that would be helpful for understanding TCM concepts and decision-making.

*Web Data*. The web data came from Web page data and Web data sets. Large masses of Web pages contained almost all types of TCM data. The online data sets were part of the standard data sources for IE, including data sets from various data integration and search platforms such as PubMed and the National Center for Biotechnology Information (NCBI) and Chinese health websites.

*Patent Data*. TCM patents often contain descriptions of drug efficacy, composition, processing methods, etc. More specifically, the extracted information included disease, syndromes, symptoms, herbal medicine, prescriptions, drug regimens, etc. The patent data always come from patent searching system or intellectual property websites.

#### 3.1.2. Extracted Information

Based on Figure 4, we further reorganized the IE tasks of TCM. It should be observed that three studies performed both entity (or term) recognition and relation extraction tasks. We were surprised that only nine studies featured relation extraction as an objective, suggesting that research on relation extraction in the field of TCM is insufficient. Remarkably, all the articles reported the data sources, and 44 reported a specific number (total words or the number of records/books). Most of the IE tasks did not focus on a particular disease or disease category (*N* = 45) but used comprehensive text as the data source. Some studies (*N* = 4) carried out extraction work from text data for a type or category of disease.

In this systematic review, we classified the information extracted from the selected studies into extracted entities and extracted relations.

*Extracted Entities*. We identified 43 studies on entity recognition (or term extraction) (*N* = 38), attribute extraction (*N* = 3), and section (or unit) extraction (*N* = (2) tasks for TCM free-text data. Recognition tasks were divided into word-level recognition work and section-level recognition work. (1) The extracted concepts of word-level recognition involved “symptom” (*N* = 24), “drug” (or medicine) (*N* = 19), “disease” (*N* = 12), “syndrome” (*N* = 7), “formula” (or prescription) (*N* = 10), “body structure” (*N* = 3), “tongue-like” (*N* = 4), “pulse” (*N* = 4), “pathogenesis” (*N* = 2), “department” (*N* = 2), “dose” (*N* = 3), “names of people” (*N* = 2), “place” (*N* = 2), “sign” (*N* = 2), “meridian” (*N* = 1), “temporal fact” (*N* = 1), “color” (*N* = 1), “quantity” (*N* = 1), “disease location” (*N* = 1), “frequency” (*N* = 1), etc. The attributes extracted (*N* = 3) contained patented drug attributes [25], disease attributes [26], and attributes of herbal medicine entities and prescriptions [27]. Among these word-level extracted entities, Zhang et al. [28] and Xu H. et al. [29] made more fine-grained division of the entities when annotating electronic health notes, which provided good demonstrations of fine-grained entity recognition. (2) Section-level entities included the clinical record unit and the sections on subjects, methods, and results [30, 31]. As the unit or section extraction is carried out on unstructured narratives, it is also classified as IE work.

*Extraction Relations*. The extracted relations involved in nine reports were the “herb-disease” relation (*N* = 4), “herb-syndrome” relation (*N* = 3), “formula-syndrome” relation (*N* = 3), “formula-disease” relation (*N* = 3), “syndrome-disease” relation (*N* = 3), “effect” relation (*N* = 1), “good for” relation (*N* = 1), “bad for” relation (*N* = 1), “same” relation (*N* = 1), “different” relation (*N* = 1), “related” relation (*N* = 1), “disease-treatment” relation (*N* = 1), “healthcare” relation (*N* = 1), “herbal-chemical” relation (*N* = 1), “therapeutic” relation (*N* = 1), and “causal” relation (*N* = 1).

### 3.2. Challenges in Extracting Information from TCM Text Data

The challenges or difficulties discussed in most papers were related to the content of TCM data and IE methods. Taking into account the domain particularity of the TCM text data, we synthesized the challenges of IE in the field of TCM into the following four points:

1. TCM texts are always unstructured and lack gold standard corpora. Chinese expressions are formed by continuous Chinese characters without spaces, and the boundary of entities is not clear. Besides, owing to the domain-specific presentation form, TCM narratives are more difficult to process than public Chinese free text [32]. Therefore, the participation of TCM experts is often indispensable to the labeling work, which increases the difficulty and cost of constructing the corpora. At present, the published corpora have a coverage rate of current semantic lexicons and the ambiguities of the lexicon words. In fact, there is still a lack of gold standard annotated data sets in the TCM field, which hinders the development of IE technologies.
2. Most of the unstructured text data in the TCM domain are nonstandardized. The contents of TCM records are always in old Chinese language and have various writing styles, as they were accomplished by different writers. Different doctors have their own social and knowledge backgrounds and different experiences, resulting in abundant nonstandard descriptions of ancient and modern TCM free-text data. For instance, “气虚”, “气弱”, and “气不足” all express the pathogenesis of Qi deficiency. “头痛”, “头疼”, and “巅顶痛” all refer to headache symptoms. Consequently, TCM always contains a hundred contending schools (TCM academic schools refer to the different divisions within the TCM clinical practice; different schools have some differences in the diagnosis and treatment methods) of thought. Clinical practitioners with historical backgrounds have different knowledge reserves and academic perspectives. This leads to the diversity of written expressions and academic views of TCM. For example, “同义词” (one meaning with different descriptions) and “一词多义” (one expression with different meanings according to the context) are very common nonstandardized phenomena in ancient and modern TCM records. Therefore, there are many differences in TCM records, including writing styles, habitual vocabularies, forms of thinking logic, and treatment habits, which bring difficulties and challenges to IE tasks.
3. There are multiple types of intricate relations in TCM data. In TCM records, there are many complicated theoretical connections in implicit form with few hints in the original text, which can only be comprehended by TCM experts. This phenomenon causes complicated relationship networks among these heterogeneous medical entities. Taking an example, the traditional Chinese Medicine Language System (TCMLS) has listed and defined 96 basic semantics types and 58 semantic relationships on the ontology top level [33]. In fact, the numbers of types of concepts and the relationships among them are far greater. Currently, more than 120,000 concepts, 300,000 terms, and 1.27 million semantic relational links have been included in the TCMLS [33]. The TCM entity relationship network is huge and complex, increasing the workload and difficulty of IE work.
4. Automatically and effectively extracting information from raw TCM text is a technical challenge. With the rapid accumulation of text data, it is impossible to construct structured data sets manually. Although more and more IE systems have been developed, the development of IE technology still needs to be explored in the face of the above three challenges. For example, how to extract information without manually annotating a large number of text data, how to extract many-to-many relations from complex TCM semantic information, and how to collect features from short texts of TCM.

### 3.3. Frameworks of Text Data Information Extraction for TCM

**Figure 6:**
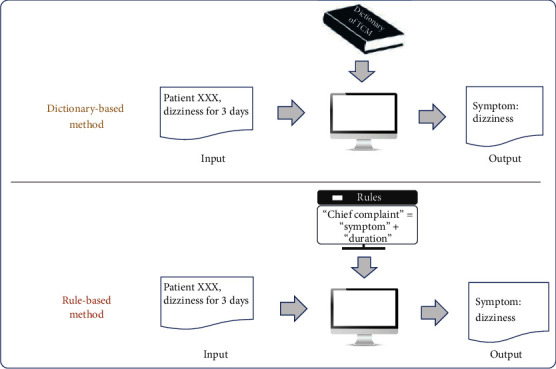
Frameworks of dictionary-based methods and rule-based methods in IE tasks.

**Figure 7:**
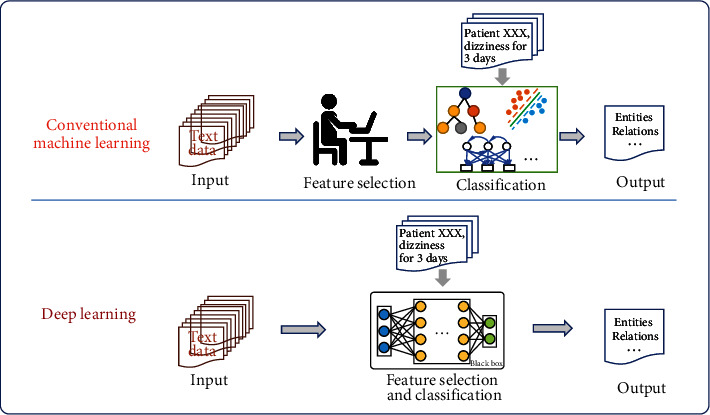
Frameworks of shallow machine learning and deep learning methods in IE tasks.

IE research methods can be divided into four categories, namely, dictionary-based methods (*N* = 7), rule-based methods (*N* = 5), shallow machine learning methods (*N* = 19), and deep learning methods (*N* = 20). The frameworks of the four types of methods are shown in Figures 6 and 7.

Dictionary-based approaches were commonly used in IE tasks in early years; they utilize existing data resources to locate entities appearing in text. Rule-based methods were frequently used in IE tasks as well. The constructed rules should fully reflect domain knowledge and language knowledge of the IE corpus. Thus, the more complete the rule set, the better the result of IE. However, rule-based methods usually require considerable manual work in constructing complicated and structured rule templates [34].

In the past decade, machine learning (ML) has become the popular method of IE with acceptable performance in the TCM domain. The breakthrough of the ML algorithm in some aspects, especially the deep learning method based on the neural network framework, has provided strong support for the progress of IE technologies. In this review, the shallow machine learning methods used to extract TCM information included supervised ML methods (*N* = 13) and the semisupervised method (*N* = 5). Supervised ML methods generally performed well in IE tasks for TCM; however, limited by the amount of training data, the semisupervised learning approach is increasingly favored by researchers.

Since 2018, deep learning methods have played an important role in IE from TCM text. A total of 18 IE tasks had used deep learning methods in this review. Among them, most of the tasks (*N* = 16) chose supervised methods to conduct extraction work. However, deep learning methods need large-scale training to prevent overfitting of the training model, and the valuable TCM clinical electronic medical records used in training are precious and few. Hence, some researchers were committed to studying how to use small amounts of labeled data to complete high-quality IE using, for example, semisupervised methods (*N* = 2) and distantly supervised methods (*N* = 2).

### 3.4. Models of Text Data Information Extraction of TCM

The algorithms of dictionary-based methods and rule-based methods were frequently used in early IE tasks. The dictionary-based IE methods relied on the artificial word library (dictionary) and mainly used string similarity algorithms, such as the matching maximum match algorithm and instance matching algorithm [35–38]. For example, Wang et al. [35] extracted symptom names exactly from segmented subsentences based on a maximum match algorithm. Zhou et al. [39] presented a domain ontology-driven semantic graph autoextraction system to discover knowledge from TCM text publications. In this work, TCMLS was used as a dictionary to extract valid domain-specific words. In the past ten years, the dictionaries used for IE have included TCM dictionaries, the TCMLS, published books of terminology of TCM, the MeSH dictionary, etc. Regarding the rule-based methods, they rely on linguistic experts to manually construct rule templates and use features including statistics, punctuation marks, keywords, indicators and direction words, location words (such as tail words), central words, etc. A common and simple form of the rule is a regular expression that uses a sequence of characters to define a search pattern [40, 41]. For instance, Yang et al. [41] extracted the temporal facts from TCM clinical records by using the regular expression method. The rule-based methods show good performance in a document where language expression has a certain pattern, such as instructions for Chinese patent medicine [25]. In addition, Cao et al. [42] combined the rules and likelihood ratio to find new terms in an iterative manner. On data from only 42 annotated health records, this method achieved a precision rate of 88.18% and a recall rate of 94.21%. Moreover, in small data sets, the rule-based approach might be superior to the feature-based approach in relation extraction tasks [43].

In the following section, we will focus on statistical-based methods, including shallow machine learning models and deep learning models. We have listed the details of model, core content, and evaluation and best performance in Table 1.

#### 3.4.1. Machine Learning Models

Shallow machine learning approaches consider IE as a classification problem and generally use supervised learning and feature engineering to obtain acceptable performance. The IE workflow of TCM using unstructured data usually consists of two main processes (as shown in Figure 7): (1) feature selection—the quality of feature selection often determines the performance of IE. In this review, the extracted features included the position feature, weight feature, counting feature, part-of-speech feature, literal feature, category feature, frequency of keywords, etc.; and (2) training or construction of a classifier based on the selected features followed by classification of new inputted unstructured data by the trained classifier. Among the 19 studies using shallow ML methods, the classifiers included decision tree (DT) [30, 43], naive Bayes (NB) [53], support vector machine (SVM) [27, 40, 51, 53], hidden Markov model (HMM) [19, 47], maximum entropy Markov model (MEMM) [19, 47], conditional random fields (CRF) [19, 30, 45–50, 53], heterogeneous factor graph [20], other NLP tools [67], etc. The unique tree structure of DT enables it to deal with continuous sequence data in IE tasks, but it ignores the correlation between attributes and easily causes overfitting results. The NB algorithm is not sensitive to missing data due to its classification stability, which is very critical in text tasks. However, its assumption of attribute conditional independence is also insensitive to the correlation between attributes. SVM is one of the most popular models for shallow machine learning classification tasks with strong advantages, and its unique kernel functions solve a wide range of classification problems. However, due to the application of kernel function, SVM is very slow in large-scale data processing, and the selection of different kernel function is sensitive to missing data. HMM contains observation sequences and hidden state sequences, which can be used for word segmentation according to different contexts by capturing potential real-time state relationships between words. However, since HMM focuses less on the context contributed, it needs to raise a certain order, which leads to the huge consumption of time and space resources in the word segmentation task of a large number of training data. The MEMM integrates the advantages of HMM and the maximum entropy model (MEM) into a production model. The model allows the state transition probability to depend on the nonindependent features in the observation sequence, which is similar to the introduction of context information by HMM. However, because it is based on the improvement of the Markov chain, it is easy to fall into the same local optimal solution as the Markov chain in the convergence process. In the process of convergence, it is easy to fall into local optimal solution. CRF is further optimized on this basis to obtain the global optimal solution by finding the joint probability for the whole sequence. To obtain more information about the sequence, various feature templates are defined to fully utilize the contextual information provided in the text. CRF-based recognizers achieved outstanding performance in different sequence annotation tasks of entity extraction [19, 47].

In order to reduce the cost and demand of manual labeling, Cai et al. [44] used semisupervised learning combined with the rule extraction method to select the window patterns of size 3 to capture the candidate words and divide them into incorrect word, correct word, and modified word, and the human-computer interaction information is added in the cycle process. In the bootstrapping process, artificially optimized selection can change the vocabulary to the drug vocabulary set. Due to the semantic ambiguity of the initial correct word seed set, artificial word judgment was added at the beginning of the semisupervised learning cycle to control the correct words seed set to prevent semantic drift. It reduces the demand for labeling data and improves the accuracy of drug vocabulary extraction from 0.682 to 0.734. Zhang et al. [17] used the method of distant supervision, in which an optimized reverse annotation method based on “Tie or Break” and the existing Chinese medicine vocabulary dictionary set are used to solve the problem of nonentity interference existing in the original annotation. The recall rate and *F*1-score index of this model in the annotation of “Complete Library of Chinese Named Motions in Past Dynasties” are ahead of other models, and the algorithm may have better room for improvement. Deng et al. [56, 57, 60] proposed a semisupervised learning method, using a small amount of data as an annotation for training, constructing a data set of first-hand Chinese herbal medicine names as the initial data set of the semisupervised learning loop, a serialized initial word segmentation, word length, pause words, valid words, and word structure as species different classifiers. The co-training method to extract disease names, medicine names, and four-character medication effect phrases from TCM patent text data.

#### 3.4.2. Deep Learning Models

With strong representations of data, better performance, and less feature engineering, deep learning methods have become the most popular method of IE and achievements have been reported. A key factor in introducing deep learning algorithms to NLP is the pretrained language model used [68], such as Word2vec or bidirectional encoder representations from transformers (BERT). Note that the BERT model is a classic and state-of-the-art language representation model of NLP technique [69], which is able to predict masked information from the context. In this review, some studies reported that the application or combination of BERT could significantly improve the result of entity recognition or relation extraction [18, 58, 59, 61]. In the last decade, the proposed deep learning models for IE tasks include BERT-convolutional neural network (CNN) [18], convolutional neural network with segment attention mechanism (SEGATT-CNN) [63], K-nearest neighbor (KNN) [53], long short-term memory (LSTM) [52, 53], bidirectional long short-term memory (BiLSTM) [17], structural BiLSTM [31], LSTM-CRF [54, 58], BiLSTM-CRF [22, 28, 55, 58, 62, 64], BERT-BiLSTM-CRF [59, 61, 66], graph neural networks [21], and a nested NER model based on LSTM-CRF [29]. Among the above-mentioned models, the “BiLSTM-CRF” and “BERT-BiLSTM-CRF” have become popular deep learning models because of their good extraction performance: the BiLSTM model can capture more context information than the LSTM model.

### 3.5. Evaluations of Information Extraction from TCM Text Data

With the exception of 10 papers that did not mention evaluation, the remaining 39 papers reported one or more evaluation metrics such as precision (or positive predictive value), recall (or sensitivity), *F*-measure (harmonic mean of recall and precision), accuracy, or AUC (area under curve). The details of evaluation metrics are shown in Table 2. Of the 39 studies that reported evaluation metrics, 28 featured true comparative evaluations. Comparisons were made between the proposed model and other models developed as part of the study or brought forward previously. Furthermore, no trend in evaluation methods was found over time.

### 3.6. Key Findings of Text Data Information Extraction for TCM

From 2010, the IE tasks used on TCM text data had achieved some results. On the one hand, there were already plenty of types of entity and several relations extracted from six data types that have been illustrated in Figure 4. On the other hand, IE technologies in the TCM domain have also yielded some results in more recent years, especially with machine learning methods.

#### 3.6.1. Key Findings on Feature Selection and Classifiers

With regard to feature selection, Zhang et al. [51] discussed the fact that the effective feature set of entity relationship classification, the contributions of verb features, all word features of entities, word distance features, and clause features are different for different types of entity relation classification and defined five types of TCM acupuncture entities, namely disease, healthcare, treatment and healthcare methods, meridians, and medication, and four entity relationship types disease-treatment, healthcare, meridian points treatment and healthcare method, and medication treatment and healthcare method. Dictionary learning is used to obtain the types of TCM entities in the text, and SVM is used to classify the different entity relationships. Jiang et al. [48] divided each case history into five attributes—words in sentences, grammatical properties of words, words in clinical dictionaries, set words acting on adjacent contexts, and set words acting at a distance—and finally found that the “grammatical property of words” is the best labeling feature among all individual labeling features and the multicombination of labeling features can improve term recognition with the CRF model constructed by local state features and local marker features for text mining in medical cases of TCM.

Regarding the classifier, Wang et al. [19] considered the signal-to-noise problem of the chief complaint as a sequence labeling problem and proposed an improved sequence labeling strategy applied to different classifiers based on certain empirical reasons. And they found that the discriminative models (MEMM and CRF) are more suitable for the symptom name recognition task than the generative model (HMM), as MEMM and CRF with useful features can achieve higher labeling performance than HMM. Liang et al. [49] proposed a novel cascade-type Chinese medication entity recognition approach, which integrated the sentence category classifier from an SVM and the CRF-based medication entity recognition. Although CRF shows good potential in IE tasks, its over-reliance on context leads to difficulties in vocabulary extraction in different contexts. In addition, an SVM is added before CRF to classify the medical records into sentences containing drug names and other sentences, and then the sentences containing target extracted drugs are introduced into CRF to reduce the confusion of word meaning in context. This approach could avoid the side effects of abundant negative samples and improve the performance of the NER from Chinese admission notes. Moreover, Wan et al. [20] used co-occurring frequency, lexical context, semantic distance as prior knowledge and the transitivity of ternary closure to explore the correlation between prior knowledge. They presented a heterogeneous factor graph model that used the correlations between all types of relations to overcome difficulties by employing collective inference in the context of heterogeneous entity networks, thus greatly improving the model's ability to extract TCM relations.

#### 3.6.2. Deep Learning Methods Improve the Performance of the Model of IE

In recent years, some findings have also been obtained by using deep learning methods. In the pretraining process, the pretraining BERT model was more accurate in learning contextual features [61]. Taking into account that there were many types of complex relation existing between many heterogeneous medical entities, Ruan et al. [21] proposed a multiview graph representation learning method that adopted the heterogeneous graph convolutional network (GCN) and attention mechanism for the extraction of complex relationships among multitype entities. They first constructed a heterogeneous entity graph from the co-occurring relations and used domain knowledge to enhance the semantic information of the above graph to infer the labels of all the candidate relations simultaneously by employing node representation learning and classification. In addition, Bai et al. [63] designed a new segment attention mechanism based on CNN, adding an attention mechanism to each segment to obtain an attention vector, which is helpful to focus on the sensitive words in the segment domain for relational classification. This study demonstrates that combining the new context representation with the word-level attention mechanism can improve the ability to extract local features of convolutional neural networks. Moreover, to address the common problem of low recognition rate of rare words in the field of TCM, Jin et al. [54] proposed an effective TCMKG-LSTM-CRF model that uses the bidirectional LSTM network chain to output features into knowledge attention vector model and finally transfer the knowledge attention vector into the conditional random field to strengthen the learning ability and recognize rare words. They also used the knowledge attention vector model to retrieve candidate entities and calculate the embedded attention vector of entities to improve the performance of existing models.

#### 3.6.3. Efforts Have Been Made to Reduce the Dependence on the Annotated Corpus

Supervision-based methods always obtain acceptable performance with enough annotated training data. However, the construction of a high-quality corpus requires laborious and time-consuming labeling work. Consequently, constructing large, high-quality corpora is difficult and costly. Currently, there are few large-scale open corpora in the field of TCM, although some corpora have already been published [19, 50, 70]. In order to reduce the cost of manual labeling under supervised learning and improve the clinical extraction ability for TCM based on feature words, Liu et al. [22] put forward a semisupervised approach for extracting TCM clinical terms based on the BiLSTM-CRF model, which combined the character vector trained by the encyclopedia corpus with that trained by the TCM-related corpus. In this study, the best *F*1 value reached 78.70% on the test data set. Zhang et al. [59] presented a semi-BERT-BiLSTM-CRF model to identify TCM entities. By adding semisupervised pseudolabel learning for model training, the recognition accuracy of the BERT-BiLSTM-CRF model was effectively improved, and the manual labeling work was reduced to a certain extent. Moreover, Zhang et al. [17] proposed a method for entity recognition based on distant-BiLSTM and completed the entity recognition task under the premise of only using the entity vocabulary related to the TCM field. This method performed better than the distant-LSTM-CRF and dictionary matching method on TCM texts. In another study, Jia et al. [23] introduced an effective distantly supervised approach at the span level to extract TCM medical entities. Unlike general sequence tagging, this method utilized the pretrained language model as a text feature extractor and constructed the span representation containing inner, boundary, and context features. In addition, they designed a negative sampling strategy for the span-level model. The strategy randomly selected negative samples in every epoch, periodically filtering possible false-negative samples and finally reducing the bad influence from false-negative samples.

## 4. Discussion and Future Work

We reviewed and narrowed down over 1,234 papers to a final set of 49 reports. Our review indicated that IE for TCM has developed rapidly and has achieved some progress. At the same time, there remain many problems to be addressed in the future. The early IE tasks relied mainly on the dictionary-based method and rule-based methods. However, domain entity dictionaries were always incomplete and could not cover all entity names in practice. In addition, the rule-based methods were heavily dependent on a good grammatical knowledge base in the TCM domain, and accordingly, the completeness and reasonability of rules were hard to guarantee [71]. In recent years, IE techniques have been dominated by statistical ML methods, including shallow ML approaches and deep learning approaches. The statistical methods of IE are widely considered advanced, with good performance. Nevertheless, the portability and generalizability of clinical IE systems remain limited and challenging. Based on these challenges, tasks, and methods presented in this review, we put forward some suggestions for future research.

First, we point out that the present methods still have strong dependence on large-scale labeled training data. Obviously, the amount of case data used for training should be enriched. At present, hospitals are becoming more and more intelligent, and it is necessary to unify the standardization of various terms in the HIS system, improve the ICD standard of traditional Chinese medicine, and obtain more and more standardized electronic medical record data. Even if the model is pretrained, the machine learning algorithms used in IE tasks are mostly conditional random fields, variants of BERT and LSTM. The few-shot learning or meta-learning technology will bring hope for the solution of this problem [72–74]. In the TCM domain, there are few publicly available corpora of TCM at present, which hinders the development of IE technology in the field of TCM. As a result, new IE tasks often require reconstruction of corpora and training of new models, resulting in lack of portability and generalizability of the models. One potential solution to this problem is to construct gold standard data sets with consensus in the field of TCM. On the basis of this, it will be helpful if more evaluation conferences of TCM IE tasks are organized. Another promising solution is developing new methods that use very small amounts of labeled data or perform without labeled data to achieve higher performance, such as meta-learning and few-shot learning technologies. Meta-learning, also known as “learning to learn”, aims to address the problem of data limitation when learning new tasks. Few-shot learning brings AI technology closer to human intelligence. Currently, meta-learning and few-shot learning have been applied in classification and target detection [75]. In the field of TCM text processing, they also have positive prospects and opportunities.

Second, there are still many concepts and relations that have not been enrolled in previous research. Therefore, the extraction of more concepts and complex relationships of TCM is an important research direction in the future, especially the implicit relations, such as the relationships among five elements (including generation, restriction, subjugation, and reverse restriction) and the compatibility relationships of Chinese herbal medicine [76]. It remains an important task to extract implicit relations directly from unstructured TCM text, which are implicitly represented in the low-dimensional and semantic space [77], as there is no relational word to prompt the type of the relation. For instance, in the record “the kidney is injured by wind evil, manifested as fast and unclear enunciation”, “is injured by” suggests a pathogenic relationship between the kidney and wind evil, while there is no cue word in the sentence to express the relationship between wind evil and the symptom “fast and unclear enunciation”. For this reason, we appeal to future research to focus on extraction of implicit relations through the cooperation of TCM experts and computer technicians.

**Figure 8:**
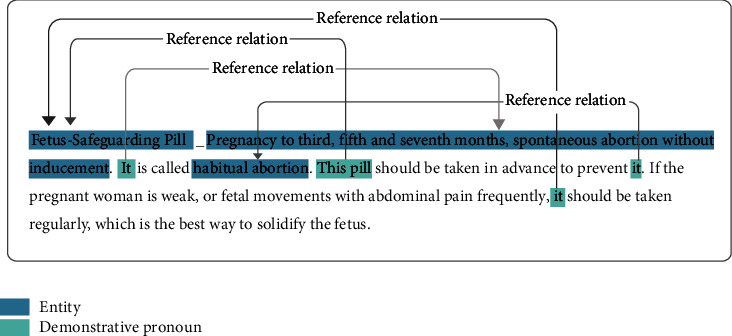
Example of the reference relations in the ancient TCM literature.

Among the six types of TCM text data, the ancient literature is a special one. It always consists of many short sentences with concise and obscure semantics, which contain enriching experience in the diagnosis and treatment of TCM [78]. For example, complex anaphora relations exist commonly in TCM ancient literature (as shown in Figure 8), and the analysis of the demonstrative pronouns and the intended referent will facilitate a deeper understanding of the meaning of the literature. However, taking the sentence in Figure 8 as an example, the demonstrative pronouns “it” and “this pill” with different syntactic positions all correspond to different contents. At the same time, the first “it” refers to the symptom “abortion without cause or reason at three, five, seven months of pregnancy”, and another “it” refers to the disease “abortion”. Obviously, the annotation of reference relations requires a sound understanding of the words, the sentences, and the context. Hence, it is challenging and meaningful to extract entities and relations from ancient literature. In future research, we expect more exploratory work on ancient literature, such as part-of-speech tagging, semantic analysis, and relation extraction.

Third, we found that most of the extracted information was coarse-grained; however, in TCM narratives, nested phenomena (entities inside an entity) are ubiquitous. For example, the syndrome “liver depression and spleen deficiency” also contains the location “liver”, “spleen”, and the nature “depression”, “deficiency”. Therefore, we hope that future studies extract entities and relationships at the fine-grained level. In this review, only one report [28] had adopted a nearly fine-grained labeling approach, in which most of the symptom entities had been disassembled into a finer one. For example, the symptom “pale complexion” was annotated as “complexion” and its attribute “pale”. In a fine-grained annotation, the coarse-grained entities will be divided into finer subcategories until no further divisions can be made. Thus, more information will be captured if fine-grained extraction is performed.

Last but not least, although deep learning methods have obtained excellent performance, the training processes are usually difficult to explain. Interpretable research on IE from TCM is still in the initial stage and will be the trend of future research. In the real-world process of clinical diagnosis and treatment, wrong decisions can have very serious consequences and will not be widely accepted. As fundamental work, the quality of extracted information will affect the performance of high-level tasks such as automatic syndrome differentiation and diagnosis and automatic prescription. Therefore, it is essential to explain the results of IE from a more detailed and representational perspective. Regarding the explicability, in August 2016, DARPA (the US Defense Advanced Research Projects Agency) proposed an explainable AI program [79]. Following this, in 2017, the NIPS (Neural Information Processing Systems) working group launched a heated discussion on the issue of whether interpretability is necessary in machine learning [80]. This discussion emphasized the importance and necessity of interpretability in the healthcare industry. For the TCM clinical domain, explanations are essential in allowing users to understand, trust, and effectively manage these AI experts [81]. With this background, explainable IE technology has attracted some attention and been explored. Taking an example, Zhang et al. [65] proposed an attention-based model to extract the semantic information on multiple aspects for the extraction of Chinese medical relations by a multihop attention mechanism. In this study, the weighting mechanism assigned weight to each word in the sentence to increase the interpretability of neural networks. Interpretability will provide more possibilities for clinical research and application of IE in the field of TCM.

There are some limitations to this review. The search strings and databases selected in this review might not be comprehensive and may have introduced bias into the review. Additionally, the review was limited to articles written in English. Articles written in other languages, such as Chinese, would also provide valuable information. Furthermore, this study focused on the tasks, challenges, and methods of IE in TCM domain; consequently some other information was not summarized in this review, such as the system development language, demographic characteristics of clinical records, and citation counts.

## 5. Conclusions

Our analyses showed that IE from TCM text data has improved over the past decade. However, the extraction of TCM text still faces some challenges involving the lack of gold standard corpora, nonstandardized expressions, and multiple types of relation. In the future, IE work should be promoted by extracting more existing entities and relations, especially the implicit relations in modern clinical records and ancient literature, constructing gold standard data sets with consensus in the field of TCM, and exploring the IE methods based on a small amount of labeled data, such as few-shot learning and meta-learning. Furthermore, fine-grained and interpretable IE technologies are necessary for further exploration. In addition, owing to the particularity of the TCM text content, more cooperation is needed between experts in the field of TCM and computer experts. Experts in ancient Chinese linguistics will be extraordinarily helpful in annotation work if the IE work is conducted on ancient literature.

## Figures and Tables

**Table 1:** Details of the statistical-based methods, core content, evaluation and best performance of IE systems.

Author, year	Method	Method type	Supervised type	Core content of extracted TCM information	Evaluation and best performance
Zhu W et al. [27], 2010	SVM	Machine learning	Supervised	Attributes of herbal medicine entities and prescriptions, including medicine application, medicine class, medicine dosage, medicine property, prescription usage, prescription attend illness, and prescription function	Medicine application: *P* = 99.6%, *R* = 98.3%; medicine class: *P* = 98.1%, *R* = 99.3%; medicine dosage: *P* = 91.4%, *R* = 93.9%; medicine property: *P* = 84.8%, *R* = 89.5%; prescription usage: *P* = 97.9%, *R* = 92.7%; prescription attend illness: *P* = 85.1%, *R* = 88.7%; prescription function: *P* = 83.6%, *R* = 92.5%
Zhu W et al. [40], 2011	SVM and regular expression	Combine the machine learning and rule-based method	Supervised	Symptom, diagnosis	Not mentioned
Cai D et al. [44], 2012	Bootstrapping approach	Machine learning	Semisupervised	Prescriptions, drugs	*F* value of prescription and drugs are 74.9% and 90.6%, respectively
Zhu W et al. [30], 2013	Decision tree and CRF	Machine learning	Supervised	Irrelevant information, basic information, multiclass information, symptom information, prescription information, and result information	Paragraph (unit) identification: *P* > 84%; information extraction: *P* > 80% (except for a book)
Feng L et al. [45], 2014	CRF and bootstrapping	Machine learning	Supervised	Clinical phenotype	Not mentioned
Wang Y et al. [19], 2014	HMM/MEMM/CRF	Machine learning	Supervised	Symptom	The best *F*-measure achieved by CRF reaches 95.12% (*P* = 94.77% and *R* = 95.48%)
Liu H et al. [46], 2015	CRF	Machine learning	Supervised	Symptoms and pathogenesis	Symptoms: *P* = 84.02%, *R* = 81.10%, *F*1 = 82.53%; pathogenesis: *P* = 86.40%, *R* = 84.65%, *F*1 = 85.52%
Jiang Q et al. [47], 2016	CRF/HMM/MEMM	Machine learning	Supervised	Symptoms, signs, TCM diagnosis, Chinese medicines (drug), prescriptions, TCM syndrome type, etc.	CRF : *P* = 90.57%, *R* = 85.85%, *F* = 88.15%; HMM = 86.29%, *R* = 79.92%, *F* = 82.98%
Jiang Q et al. [48], 2016	CRF	Machine learning	Supervised	Symptoms or signs, TCM diagnosis, TCM syndrome type, Chinese medicines (drug), and names of TCM prescriptions	With all features: *P* = 89.74%, *R* = 82.9%, *F* = 86.18%
Wang J et al. [43], 2016	Decision tree/rules	Machine learning and rule-based method	Supervised	Effect relation and conditional effect relation	Rule-based approach: *F* = 46%; feature-based approach: *F* = 41%
Wan H et al. [20], 2016	Heterogeneous factor graph model (HFGM)	Machine learning	Semisupervised	Herb-syndrome relations, herb-disease relations, formula-syndrome relations, formula-disease relations, and syndrome-disease relations	*P* = 90.39%, *R* = 86.98%, *F*1 = 88.56%
Liang J et al. [49], 2017	CRF	Machine learning	Supervised	TCM drug name	Recognition of traditional Chinese medicine drug names: *P* = 94.2%, *R* = 92.8%, *F* = 93.5%
Ruan T et al. [50], 2017	CRF	Machine learning	Supervised	Symptoms, departments, disease, medicines, and examinations	Precision of TCM symptom is 93.26%
Sun S. et al. [51], 2017	SVM	Machine learning	Supervised	Disease-treatment relation, healthcare relation, meridian points treatment and healthcare method relation, and medication treatment and healthcare method relation	The *F*-measure of entity relation classification model of disease-treatment, healthcare, meridian points treatment and healthcare method, and medication treatment and healthcare method reached 93.25%, 87.19%, 86.57%, and 84.57%, respectively
Zhang H. et al. [52], 2018	Short-text LSTM classifier (STLC)	Deep learning	Supervised	Person name	*F*1 = 92.75%
Chi Y. et al. [53], 2018	CRF for entity extraction; SVM, naive Bayes (NB), LSTM, and KNN for relation extraction	Machine learning and deep learning	Supervised	Entities: food material, dish, nutritional element, symptom, and crowd; relations: “good for” or “bad for” relationships between “food material” and “symptom”, and “same”, “related”, or “different” relationships between two “food material” entities	Concept extraction and relationship recognition were all above 85%
Chen T et al. [18], 2019	Combine BERT with a one-dimensional CNN to fine-tune the pretrained model	Deep learning	Supervised	Herb-syndrome relation, herb-disease relation, formula-syndrome relation, formula-disease relation, and syndrome-disease relation	The “1d-CNN fine-tuning” approach: *P* = 95.18%, *R* = 90.61%, *F*1 = 92.82%
Jin Z et al. [54], 2019	TCMKG-LSTM-CRF	Deep learning	Supervised	Medicine, alias, prescription, pieces, disease, symptom, syndrome, meridian, property, flavor, and function	*F* = 85.41%
Song B et al. [55], 2020	BiLSTM-CRF	Deep learning	Supervised	Symptom	*P* = 76.80%, *R* = 72.11%, *F*1 = 74.38%
Deng N et al. [56, 57], 2020	Co-training-based method	Machine learning	Semisupervised	Four-character medicine effect phrases	In each iteration, the extraction accuracy is all above 97%
Deng N et al. [57], 2020	Serialized co-training method	Machine learning	Semisupervised	Medicine names	Precision is 98.6% when the number of patent texts reaches 3000
Feng L et al. [58], 2020	BiLSTM-CRF, lattice LSTM-CRF, BERT	Deep learning	Supervised	Symptom	The BERT model: *P* = 89.94%, *R* = 88.27%, *F*1 = 89.10%
Liu L et al. [22], 2020	BiLSTM-CRF	Deep learning	Semisupervised	Traditional Chinese medicine, symptoms, patterns, disease, and formulas	*F*1 = 78.70%, *P* = 77.56%, *R* = 79.87%
Zhang M et al. [59], 2020	BERT-BiLSTM-CRF	Deep learning	Semisupervised	Symptoms, syndromes, treatment, Chinese medicine, prescriptions, pulse, tongue, and efficacy	Accuracy = 81.24%, *R* = 80.84%, *F*1 = 81.04%.
Deng N et al. [60], 2020	Serialized co-training method	Machine learning	Semisupervised	Disease name	Not mentioned
Qu Q et al. [61], 2020	BERT-BiLSTM-CRF	Deep learning	Supervised	Symptoms, disease names, time, prescription names, and drug names	The best recognition effect on drugs, with an *F* value as high as 88.79%
Ruan C et al. [21], 2020	Multiview graph model to extract relation (MVG2RE)	Deep learning	Supervised	Herb-syndrome, herb-disease, formula-disease, formula-syndrome, and syndrome-disease	*P* = 86.38%, *R* = 81.42%, *F*1 = 84.07%
Zhang D et al. [17], 2020	BiLSTM	Deep learning	Distantly supervised	Chinese medicine, symptom, medicine prescription, dose, tongue-like, and pulse	*P* = 73.06%, *R* = 66.75%, *F* = 69.76%
Zhang H et al. [28], 2020	BiLSTM-CRF	Deep learning	Supervised	Chief complaints (symptom name, symptom duration, accompanying symptom, etc.); pathological history (symptom name, symptom cause, etc.); personal history (place of birth, smoking history, drinking history, etc.); and TCM diagnosis (tongue quality, furred tongue, tongue body, pulse, etc.)	Not mentioned
Zheng Z et al. [62], 2020	BiLSTM-CRF	Deep learning	Supervised	Syndrome, disease, symptom, prescription, therapy, herb	*F*1 > 97%
Zhou S et al. [31], 2020	Structural bidirectional long short-term memory (SLSTM) model	Deep learning	Supervised	Subjects, methods, and results	SLSTM model achieves close to 90% performance in precision, recall, and *F*1-measures
Bai T et al. [63], 2021	SEGATT-CNN combined with different classifiers	Deep learning	Supervised	Entity: disease, herb names; relation: herbal-disease relation, herbal-chemical relation	Herbal-disease relation: SVM combined SEGATT-CNN: *P* = 95.43%, *R* = 92.92%, *F* = 94.15%, AUC = 95.13%, accuracy = 95.69%
Deng N et al. [64], 2021	BiLSTM-CRF	Deep learning	Supervised	Herb name, disease name, symptom, therapeutic effect, and nonentity	*P* = 94.63%, *R* = 94.47%, *F*1 = 94.48%
Jia Q et al. [23], 2021	Span-level distantly supervised NER approach	Deep learning	Distantly supervised	Symptom, medicine, prescriptions, dose, tongue-like, and pulse	*P* = 78.28%, *R* = 76.52%, *F*1 = 77.34%
Zhang T et al. [65], 2021	Multihop self-attention mechanism + BiLSTM	Deep learning	Supervised	Therapeutic relation and causal relation	Therapeutic relation: *P* = 88.65%, *R* = 98.23%, F1-score = 93.19% and causal relation: *P* = 69.23%, *R* = 78.26%, *F*1 = 73.47%
Xu H et al. [29], 2021	Nested NER model based on LSTM-CRF	Deep learning	Supervised	Medicine, symptom, pulse, tongue, medicine prescription, dose, disease location, onset time and duration, severity, color, quantity, and frequency	*P* = 85.64%, *R* = 86.18%, *F*1 = 85.91%
Guan Y et al. [66], 2021	BERT-BiLSTM-CRF	Deep learning	Supervised	Disease, pattern, and symptom	*P* = 86.54%, *R* = 88.59%, *F*1 = 87.55%, when the size of training set is 10,000

**Table 2:** Details of evaluation metrics.

Evaluation index	Formula	Role
Precision	Precision=*TP*/*TP*+*FP*	Determine the ratio of correctly predicted positive samples
Accuracy	Accuracy=*TP*+*TN*/*TP*+*TN*+*FP*+*FN*	Determine the correct rate of all classifications
Recall	Recall=*TP*/*TP*+*FN*	Ratio of correctly predicted positive samples to the total number of real positive samples
*F*-measure	F − measure=(1+*β*^2^)precision^*∗*^recall/*β*^2^precision+recall	Examine precision and recall
AUC	Area under curve	Intuitively express the real classification ability of the model

## Data Availability

The data extracted from the included papers used to support the findings of this study are included within the supplementary information files.

## Abbreviations

IE: Information extraction
TCM: Traditional Chinese medicine
AI: Artificial intelligence
NLP: Natural language processing
PRISMA: Preferred Reporting Items for Systematic Reviews and Meta-Analyses
RANs: Resident admit notes
NCBI: National Center for Biotechnology Information
TCMLS: Traditional Chinese Medicine Language System
CRF: Conditional random fields
SVM: Support vector machine
HMM: Hidden Markov model
MEMM: Maximum entropy Markov model
DT: Decision tree
NB: Naive Bayes
BERT: Bidirectional encoder representations from transformers
CNN: Convolutional neural network
SEGATT: Segment attention mechanism
KNN: K-nearest neighbor
LSTM: Long short-time memory
BiLSTM: Bidirectional long short-term memory
AUC: Area under curve
GCN: Graph convolutional network
DARPA: Defense Advanced Research Projects Agency
NIPS: Neural Information Processing Systems.

## Supplementary Materials

Supplementary Materials Supplementary 1. PRISMA checklist. Supplementary 2. The basic information and extracted data of included papers.
