# Supplementary material for: Information Extraction from the Text Data on Traditional Chinese Medicine: A Review on Tasks, Challenges, and Methods from 2010 to 2021
Source: Evid Based Complement Alternat Med. 2022 May 13;2022:1679589. doi: 10.1155/2022/1679589 (PMC9122692; doi:10.1155/2022/1679589)
Supplement: Supplementary Materials — Supplementary 1. PRISMA checklist. Supplementary 2. The basic information and extracted data of included papers. [file 1679589.f1.zip › Appendix 2_The basic information and extracted data of each included paper.pdf]

Order number	Author	Title	Published year	Document type	Data type	Data number and source of TCM	Disease	System	Methods description (system/model / tool)	Method type	Supervised type	Core content of extracted TCM informations	NLP tasks related to information extraction	Evaluation and Best Performance	Comparative Evaluation	Comparative Evaluation	Objective	Relevant outcomes	Challenges
4	Cai D et al., 2011	A semi-supervised learning method for names of traditional Chinese prescriptions and drugs recognition	2011	CA	Ancient literature	519 medical records from "Classified Medical Records of Distinguished Physicians" and " Classified Medical Records of Distinguished Physicians Continued"	Not specified	None	Bootstrapping Approach to Recognize Names of Traditional Chinese Prescriptions and DrUgs using Semantic Knowledge	Machine learning	Semi-supervised	Prescriptions, drugs	Entity extraction	F-Value of prescription and drugs are 74.9% and 90.6% respectively	√	Compared with no human-computer interactions during recognition process	Recognize names of Traditional Chinese prescriptions and drugs in un-annotated ancient medical literatures.	In this paper, a method named Barpidusk, which uses simple context features instead of lexical and syntactic knowledge, was proposed. In the method, iteration process was simplified; and ambiguities or mistakes brought by pre-processing of corpus were avoided. Furthermore, human-computer interaction is added to Bootstrapping for generating a new framework, within which the semantic drift was resolved and system efficiency was improved.	Due to the diversity of ancient medical literatures, low coverage rate of current semantic lexicons and the ambiguities of the lexicon words, low recall rate and low accuracy are resulted by using only lexicons to recognize TCM terms.
10	Wang Y et al, 2014	Supervised methods for symptom name recognition in free-text clinical records of traditional Chinese medicine: An empirical study	2014	JA	Clinical records	11613 TCM clinical record collected by TCM doctors during their routine diagnostic work from April 2006 to June 2008	Not specified	None	HMM / MEMM / CRF	Machine learning	Supervised	Symptom	Entity extraction	the best F-measure achieved by CRF reaches 95.12% (Precion= 94.77% and Recall= 95.48%)	√	Comparison among HMM, MEMM, CRF	Extract symptom name from free text clinical record in machine learning methods.	The domain-specific adaptation of sequence labeling strategies is appropriate and effective, and that CRF outperforms HMM and MEMM for the SNR task.	The methods that are verified on the well-structured data cannot be directly applied to knowledge discovery in the free-text clinical records of TCM. However, manually structuring the large volume of TCM FCRs is tedious, time-consuming, and labor intensive. Hence there is an urgent need for the development of an effective method to automatically structure the free-text clinical records.
12	Jiang Q et al., 2016	Free text mining of TCM medical records based on conditional random fields	2016	CA	Clinical records	more than 600 free text of TCM medical records	Not specified	None	CRF/ HMM / MEMM	Machine learning	Supervised	Symptoms, signs, TCM diagnosis, Chinese medicines (drug), prescriptions, TCM syndrome type, etc.	Entity extraction	CRF: Precision=90.57%, Recall=85.85%, F-score=88.15%; HMM=86.29%, Recall=79.92%, F-score=82.98%	√	Comparisons among CRF, HMM and MEMM	Aim to give an extracted method with text mining based on conditional random fields.	CRF is more appropriate and precise than HMM, ME and MEMM on clinical terms recognitions in TCM medical records.	How to extract medical terms from TCM medical records based on conditional random fields.
13	Jiang Q et al., 2016	Multi-combined features text mining of TCM medical cases with CRF	2016	CA	Clinical records	about 600 TCM medical cases obtained from Traditional Chinese Medical Hospital of Guangdong Province	Not specified	None	CRF	Machine learning	Supervised	Symptoms or signs, TCM diagnosis, TCM syndrome type, Chinese medicines (drug), and names of TCM prescriptions	Entity extraction	With all features: P=89.74%, R=82.9.%, F=86.18%	√	Compared with different combined labeling features	In order to improve accuracies of recognitions, CRF model is used for terms recognitions in TCM medical cases with multi- labeling features.	The results show that, "grammatical property of words" is the best labeling features in all individual labeling features. Multi-combined features have higher scores than individual labeling features on improving clinical terms recognition. The combined mode of grammatical property of words", "words in sentence", and "words in clinical dictionary" may be the most suitable labeling features. Multi-combined labeling features can improve term recognition with CRF model for text mining in TCM medical cases.	How to extract the data and terms automatic and intelligently, and classify into form a database.
14	Wang J et al., 2016	Relation extraction from Traditional Chinese Medicine journal publication	2016	CA	Journal publications	31 pieces of TCM journal publication	Not specified	None	Decision Tree / rules	Machine learning; rule-based method	Supervised	Effect relation, conditional effect relation	Relation extraction	Rule-based approach: F-score=46%; feature-based approach: F-score=41%	√	Comparison between the rule-based approach and The feature-based approach	Extract relation from traditional Chinese medicine journal publication.	The rule-based approach excels the feature-based approach which may be due to the small size of the dataset at hand. This research introduces and compares two extraction approaches, in which also address some of the more Chinese-specific NLP problems, such as word segmentation andflexible syntactic structure	How to extract relation from TCM text which is made more difficult by the Chinese language. The Chinese language differs significantly from English, both in lexical representation and grammatical structure. These differences lead to problems in Chinese NLP, such as word segmentation and flexible syntactic structure.
15	Wan H et al, 2016	Extracting relations from traditional Chinese medicine literature via heterogeneous entity networks	2016	JA	Journal publications	106150 abstracts in published articles on TCM from a Chinese publication database	Not specified	None	Heterogeneous factor graph model (HFGM)	Machine learning	Semi-supervised	Herb-syndrome relations, herb-disease relations, formula-syndrome relations, formula-disease relations, syndrome-disease relations.	Relation extraction	Precision=90.39%, Recall=86.98%, F1=88.56%	√	Compared with a traditional SVM classifier	Collectively and globally extract multiple types of relations from the entire corpus of TCM literature.	Heterogeneous factor graph model (HFGM) based on heterogeneous entity networks significantly improved the ability to extract relations from TCM literature.	The main challenge of relation extraction from TCM data is the complexity of the TCM systemitself. Multiple types of interwoven relations can exist between tens of thousands of heterogeneous TCM entities.
16	Liang J et al, 2017	A Novel Approach towards Medical Entity Recognition in Chinese Clinical Text	2017	JA	Clinical records	972 admission notes from Second Affiliated Hospital Zhejiang University School of Medicine	Not specified	None	CRF	Machine learning	Supervised	TCM drug name	Entity extraction	Recognition of traditional Chinese medicine drug names: precision=94.2%, recall=92.8%, F-measure=93.5%	√	Compared with the baseline system which used the maximum matching algorithm and professional drug dictionaries (dictionary-based methods)	Proposed a new cascade-type approach towards medical entity recognition in Chinese.	The proposed approach avoids the side effects due to abundant negative samples and improves the recognition performance of drug named enti ties in the logographic (Chinese) descriptions	Much less work has been done on nonstructural texts that are written in Chinese, or in the setting of differentiation of Chinese drug names between traditional Chinese medicine and Western medicine.
19	Zhang H et al., 2018	TCMEF: A TCM Entity Filter Using Less Text	2018	CA	Web data	7500 TCM related entity titles from Baidu Baike and Hudong Baike	Not specified	TCM entity filter (TCMEF)	Short Text LSTM Classifier (STLC)	Deep learning	Supervised	Person name	Entity extraction	F1 score of TCMEF=92.75%	√	Compared with general word based short text classification algorithms and Latent Dirichlet Allocation based model	Propose a three-phase filtering framework for TCM entities	The three-phase fifiltering framework for TCM entities uses the stroke features of Chinese characters to recognize the TCM short text titles and make individual identifification for person names. It saves network, storage and computing costs for online entity filtering since we only have to download pages and analyze rich text for less than 10% of the entities.	The entities in text style and character shape in TCM titles are often shorter than 20, many of them are even shorter than 10 characters. It's hard for general short text classification algorithms to collect enough features for these titles.
21	Chi Y et al., 2018	Knowledge Management in Healthcare Sustainability: A Smart Healthy Diet Assistant in Traditional Chinese Medicine Culture	2018	JA	Web data	China Food Composition and three Chinese health websites. Training:4000; test: 1000	Not specified	None	CRF for entity extraction; SVM, naive Bayes (NB), LSTM, and KNN for relation extraction	Machine learning; deep learning	Supervised	Entities: food material, dish, nutritional element, symptom, and crowd; relations: "good for" or "bad for" relationships between " food material" and "symptom", and "same", "related" or " different" relationships between two "food material" entities	Entity extraction; relation extraction	concept extraction and relationship recognition were all above 85%.	√	Compared among SVM, NB, LSTM, KNN	To help people retrieve and learn healthy diet knowledge more efficiently and comprehensively, this paper designs a knowledge graph to integrate healthy diet information on the internet and provides a semantic retrieval system.	In the knowledge graph, five main concepts are defined, including food material, dish, nutritional element, symptom, and crowd, as well as the relationships among them. By using the system, users learn healthy diet knowledge more quickly and comprehensively and they are more inclined to have balanced diets.	The data regarding healthy diets available on the internet is increasing rapidly and is distributed on multiple sources. It is time-consuming for users to learn about healthy diets on the internet: they need to search data on multiple platforms, choose and integrate information, and then understand what they have learned.
22	Chen T et al, 2019	A general approach for improving deep learning-based medical relation extraction using a pre-trained model and fine-tuning	2019	JA	Journal publications	106150 abstracts from papers published in 114 most popular Chinese TCM journals	Not specified	None	Combine BERT with a one-dimensional convolutional neural network (1d CNN) to fine-tune the pre-trained model	Deep learning	Supervised	Herb-syndrome relation, herb-disease relation, formula-syndrome relation, formula-disease relation, syndrome-disease relation	Relation extraction	The '1d-CNN fine-tuning' approach: Precision=95.18%, Recall=90.61%, F1-measure=92.82%	√	Compared with SVM, iterative SVM, HFGM, 1d-CNN general embeddings, 1d-CNN TCM embeddings, BERT with pre-trained model	Propose a pre-trained model and a fine-tuning technique to improve these approaches without additional time-consuming human labeling.	The '1d-CNN fine-tuning' approach achieves the best performance on four of the five relation types.	Most of the current deep learning approaches for medical relation extraction require large-scale training data to prevent overfitting of the training model.
23	Gong F et al, 2019	On building a diabetes centric knowledge base via mining the web	2019	JA	Web data	7703 instances and 96041 edges from eight prevalent vertical portals on health care in China	Diabetes	None	Instance matching algorithm	Dictionary-based method		Traditional Chinese medicine	Entity extraction	The accuracy of overall knowledge base is 98.1%	√	Compare with instance matching with pre-processing	Constructing a high-coverage knowledge base of a specific disease to support the above extraction tasks called KB-based Extraction	Introduced an approach to constructing diabetes knowledge base.	To support the analytical procedures, researchers and analysts expend a mass of labor cost to collect experimental data, which is also error-prone. To reduce the cost and to ensure the data quality, there is a growing trend of extracting clinical events in form of knowledge from electronic medical records (EMRs)
24	Jin Z et al., 2019	Named Entity Recognition in Traditional Chinese Medicine Clinical Cases Combining BiLSTM-CRF with Knowledge Graph	2019	CA	Text book	75 modern TCM clinical cases and 75 ancient TCM clinical cases from two authoritative clinical case books and literatures in CKCEST	Not specified	None	TCMKG-LSTM-CRF	Deep learning	Supervised	Medicine, alias, prescription, pieces, disease, symptom, syndrome, meridian, property,flavour and function	Entity extraction	F-score=85.41%	√	Compared with CRF, BiLSTM-CRF, BiLSTM-CRF Pretrained	These methods cannot effectively solve the problem of low recognition rate of rare words, which is common in TCM field	Propose TCMKG LSTM-CRF model that utilizes knowledge graph information to strength the learning ability and recognize rare words. This model introduces knowledge attention vector model to implement attention mechanism between hidden vector of neural networks and knowledge graph candidate vectors and consider influence from previous word. The experiment results prove the effectiveness of our model	How to effectively solve the problem of low recognition rate of rare words, which is common in TCM field.
26	Song B et al., 2020	Incorporating Lexicon for Named Entity Recognition of Traditional Chinese Medicine Books	2020	CA	Ancient literature	376 TCM books from the Han Dynasty to the Qing Dynasty	Not specified	None	BiLSTM-CRF	Deep learning	Supervised	Symptom	Entity extraction	Precision=76.80%, Recall=72.11%, F1=74.38%	√	Compared with HMM, CRF	In order to improve the performance of NER for TCM books	Propose a method which is based on biLSTM-CRF model and can incorporate lexicon information into representation layer to enrich its semantic information. Experimental results on annotated data of "Shanghan Lun" show that this method outperforms previous models for NER of TCM books	Lexicon information and large-scale of unlabeled corpus data are not fully exploited in the previous methods.
29	Feng L et al., 2020	Research on Named Entity Recognition of Traditional Chinese Medicine Electronic Medical Records	2020	CA	Clinical records	131 electronic medical records from the Hubei Provincial Hospital of Traditional Chinese Medicin	Not specified	None	BiLSTM-CRF, lattice LSTM-CRF, BERT	Deep learning	Supervised	Symptom	Entity extraction	The BERT model: Precision=89.94%, Recall=88.27%, F1 value=89.10%.	√	Compared with BiLSTM-CRF and lattice LSTM-CRF	In order to explore the effective method of named entity recognition of TCM EMR.	This paper selects three models, BiLSTM-CRF, lattice LSTM-CRF and BERT, to recognize the symptom entities in EMR, and carries out comparative experiments. The experimental results show that BERT model has the best recognition effect about TCM EMR, the precision is 89.94%, the recall is 88.27%, and the F1 value is 89.10%.	(1) The naming entity of traditional Chinese medicine has complex characteristics and uncertain length. In addition, there are sub entities in the entity. Therefore, the work of named entity recognition in traditional Chinese medicine is more complex and more difficult than that in general fields. (2) Character-based model can avoid word segmentation errors, but it can also lose the semantic information contained in the word sequence in the context
31	Liu L et al, 2020	A semi-supervised approach for extracting TCM clinical terms based on feature words	2020	JA	Text book	*Formulas of Chinese Medicine* (28600 words)	Not specified	None	BiLSTM-CRF	Deep learning	Semi-supervised	Traditional Chinese medicine, symptoms, patterns, diseases and formulas	Entity extraction	F1-value=78.70%, Precision=77.56%, Recall=79.87%	√	Compared with the supervised method	Proposes a semi-supervised approach for extracting TCM clinical terms based on character vector.	The method reduces the cost of manual labeling and improves the experiment results based on the help of the feature word.	(1) Ancient Chinese language is extensively used in the TCM corpus which brings difficulties to TCM NER research. (2) manual labeling of supervised methods are costly and labor-intensive.
32	Zhang M et al., 2020	Traditional Chinese Medicine knowledge Service based on Semi-Supervised BERT-BiLSTM-CRF Model	2020	CA	Ancient literature	Ancient Chinese medicine books	Not specified	None	BERT-BiLSTM-CRF	Deep learning	Semi-supervised	Symptoms, syndromes, treatment, Chinese medicine, prescriptions, pulse, tongue, efficacy	Entity extraction	Accuracy=81.24%, Recall=80.84%, F1 value=81.04%.	√	Compared with CRF, BiLSTM-CRF	This paper proposes a semi-supervised method to extract entities from TCM texts.	Experiments show that TCM entity recognition accuracy of this embedded Semi-BERT-BiLSTM-CRF model reaches 81.24%, which effectively improves the TCM entity recognition accuracy and reduces the manual labeling work. The results of this research can be applied to scenarios such as auxiliary diagnosis of TCM and expert system after subsequent improvement and transformation	The existing methods are not accurate enough to solve TCM named entity recognition and require a lot of manual labeling data.
34	Qu Q et al., 2020	Named Entity Recognition of TCM Text Based on Bert Model	2020	CA	Ancient literature	A total of 32614 characters from "Treatise on Febrile Diseases"	Not specified	None	BERT-BiLSTM-CRF	Deep learning	Supervised	Symptoms, disease names, time, prescription names, and drug names	Entity extraction	The best recognition effect on drugs, with an F-value as high as 88.79%	√	Compared with BiLSTM-CRF, LSTM-CRF	Aiming at the problem of fuzzy entity recognition and less labeled data in the field of traditional Chinese medicine, a named entity recognition model based on Bert-BiLSTM-CRF is constructed and tested on the corresponding data set	By constructing the word vector through the Bert model and then using it in BiLSTM-CRF, both the accuracy rate, the recall rate, or the F value are improved.	The problem of fuzzy entity recognition and less labeled data in the field of TCM.
35	Ruan C et al, 2020	Relation Extraction for Chinese Clinical Records Using Multi-View Graph Learning	2020	JA	Clinical records	More than 100,000 paper abstracts from a Chinese publication database	Not specified	None	Multi-view graph model to extract relation (MVG2RE)	Deep learning	Supervised	Herb-syndrome, herb-disease, formula-disease, formula-syndrome, and syndrome-disease	Relation extraction	Precision=86.38%, recall=81.42%, F1-score= 84.07%	√	Compared with SVM-C, PTM, ADRN, SHR, RelExtr	Extract relation from Chinese clinical records.	Propose a multi-view graph representation learning that adopts heterogeneous GCN and attention mechanism for complex relation extraction among multi-type entities	Very limited relation extraction research has been conducted on clinical notes written in Chinese, especially TCM clinical records.
37	Yang C et al., 2020	A temporal semantic search system for traditional Chinese medicine based on temporal knowledge graphs	2020	CA	Clinical records	Classic Chinese Medicine Clinical Case Library	Not specified	None	Regular expression	Rule-based method		Temporal facts	Terms extraction	Average Precision=0.825	√	Compared with tf-idf, tf-idf+entity linking	Implement a temporal semantic search system.	Propose a new model tailored for TCM based on the temporal knowledge graph in this paper.	Current TCM-based retrieval systems cannot effectively deal with the temporal intentions of search sentences, which leads to bad experiences for users in retrieval services. Most of temporal information are ignored in the knowledge map construction.
39	Zhang D et al, 2020	Improving Distantly-Supervised Named Entity Recognition for Traditional Chinese Medicine Text via a Novel Back-Labeling Approach	2020	JA	Ancient literature	Chinese medical records of all famous doctors(100000 words)	Not specified	None	BiLSTM	Deep learning	Distantly supervised	Chinese medicine, symptom, medicine presciption, dose, tongue-like, pulse	Entity extraction	Precision=73.06%, Recall=66.75%, F1-measure=69.76%	√	Compared with Distant-LSTM-CRF and dictionary matching method	Propose a method for entity recognition based on distant supervision, and complete the entity recognition task under the premise of only using the entity vocabulary related to the TCM field.	The scheme can effectively improve the entity recognition on the basis of distant supervision.	The limitation of using machine learning methods, which in need of a large amount of manually labeled data.
43	Zhou S et al., 2021	Feature engineering vs. deep learning for paper section identification: Toward applications in Chinese medical literature	2021	JA	Journal publications	371 papers with 15057 sentences from human-curated asthma literature datasetd from CNKI	Asthma	None	Structural Bidirectional Long Short Term Memory (SLSTM) model	Deep learning	Supervised	Subjects, methods, results	Section extraction	SLSTM model achieve close to 90% performance in precision, recall, and F1-measures	√	Compared with CRF, BLSTM, HAN, CLSTM	Studied the paper section identification problem in the context of Chinese medical literature analysis, where the subjects, methods, and results are more valuable from a physician's perspective.	Design a novel deep learning model, the Structural BiLSTM model, which outperforms the traditional machine learning methods and other deep learning methods.	Due to natural language differences, the extracted features from English texts cannot be directly applied onto Chinese medical literature. Moreover, Chinese medical literature is often less structured than English literature, which makes the problem more difficult to tackle.
44	Bai T et al., 2021	Traditional Chinese medicine entity relation extraction based on CNN with segment attention	2021	JA	Web data	Annotated dataset with 4732 samples of herbs-diseases and 4666 samples of herbs chemicals constructed by PubMed	Not specified	None	SEGATT-CNN combined with different classifiers	Deep learning	Supervised	Entity: disease entity, herb names; Relation: herbal-disease relation, herbal-chemical relation,	Entity extraction, relation extraction	Herbal-disease relation: SVM combined SEGATT-CNN: P=0.9543, R=0.9292, F=0.9415, AUC=0.9513, Accurency=0.9569	√	Compared with SVM, KNN, DT, RF	In order to extract Chinese medicine-specific entity relations more reliably and efficiently from PubMed literature.	Propose a novel architecture with an improved layer for entity relation extraction. The method has an absolute advantage and is proved to be effective in solving this problem of herbal related relation extraction.	(1) Although the deep learning technology has been applied to the relation extraction task with superiorresults, relevant works are seldom adapted to TCM area. (2) language gap exists between TCM prac tices and researches, (3) classical machine learning algorithms improve the accuracy of relation extraction primarily through complicated manual feature engineering, which constrains the potential of accuracy improvement for classical machine learning.
45	Na D et al., 2021	Named Entity Recognition of Traditional Chinese Medicine Patents Based on BiLSTM-CRF	2021	JA	Patent texts	a total of 2000 TCM patents' abstract texts from intellectual property websites	Not specified	None	BiLSTM-CRF	Deep learning	Supervised	Herb name, disease name, symptom, therapeutic effect, nonentity	Entity extraction	precision rate is 94.63%, recall rate is 94.47%, and F1 value is 94.48%	√	Compared with HMM,LSTM, BiLSTM	This study aim to design a model to fill the technical gap in NER of TCM patents.	A method combining Bidirectional Long Short-Term Memory neural network with Conditional Random Field (BiLSTM-CRF) is proposed to automatically recognize entities of interest (i.e., herb names, disease names, symptoms, and therapeutic effects) from the abstract texts of TCM patents.By virtue of the capabilities of deep learning methods, the semantic information in the context can be learned without feature engineering. Experiments show that the BiLSTM-CRF-based method provides superior performance in comparison with various baseline methods.	(1) Due to the long history and wide geographical distri bution of herbs. (2) Due to different writing habits, TCM doctors and researchers tend to express a certain disease name in languages similar with classical Chinese. (3) Due to the lack of labeled training sets of TCM patent texts, a proper training set has to be established man ually while applying machine learning method for NER.
46	Jia Q et al., 2021	Extraction of Traditional Chinese Medicine Entity: Design of a Novel Span-Level Named Entity Recognition Method With Distant Supervision	2021	JA	Clinical records	Case book of TCM compiled by the expert team of Professor Lu Zhaolin	Not specified	None	Span-level distantly supervised named entity recognition (NER) approach	Deep learning	distantly supervised	Symptom, medicine, prescriptions, dose, tongue-like, pulse	Entity extraction	Precision=78.28, Recall=76.52, F1-score=77.34	√	Compared with distant-LSTM-CRF, Novel label-back AutoNER, AdaPU, BERT-CRF.	The idea is to utilize distantly supervised method to conducted the name entity recognition (NER) task.	Propose a span-level distantly supervised named entity recognition (NER) approach to extract TCM medical entity. It utilizes the pre-trained language model, a simple multi-layer neural network as classifier to detect and classify entity. We also design a negative sampling strategy for the span-level model. The strategy randomly selects negative samples in every epoch and filter the possible false negative sample periodically. It reduces the bad influence from the false negative samples	(1) Most of TCM clinical records are unstructured text; (2) The narrative is free-style and difficult to understand for modern medical practitioners. The cost of manually structuring and maintaining free-text clinical records is very expensive; (3) lack of gold standard datasets for supervised learning methods.
47	Zhang T et al., 2021	Chinese medical relation extraction based on multi-hop self-attention mechanism	2021	JA	Journal publications	4000 Chinese medical literature abstracts	Not specified	None	Multi-hop self-attention mechanism+BiLSTM	Deep learning	Supervised	Therapeutic relation, causal relation	Relation extraction	Therapeutic relation: Precision=88.65, Recall=98.23, F1-score=93.19; Causal relation:Precision=69.23, Recall=78.26, F1-score=73.47	√	Compared with CNN, Bi-LSTM, Bi-LSTM+self-attention, Bi-LSTM +multi-head attention	Propose an attention-based model to extract the multi-aspect semantic information for the Chinese medical relation extraction by multi-hop attention mechanism.	The model could generate multiple weight vectors for the sentence through each attention step, therefore, we can generate the diferent semantic representation of a sentence, respectively. Experimental results on two Chinese medical datasets show that our model is highly effective among existing experiments, achieving state-of the-art results with little feature engineering.	(1) Complex semantic information in the sentence determines the relation between entities, the semantic information cannot be represented by one sentence vector; (2) machine learning methods rely on manual con struction of features, which is laborious and time-consuming.
48	Xu H et al., 2021	A Nested Named Entity Recognition Method for Traditional Chinese Medicine Records	2021	CA	Clinical records	4096 sentences from《中华历代名医医案全库》(Chinese medical records of all famous doctors)	Not specified	None	Nested NER model based on LSTM-CRF	Deep learning	Supervised	Medicine, symptom, pulse, tongue, medicine prescription, dose, disease location, onset time and duration, severity, color, quantity, frequency	Entity extraction	Precision=85.64, Recall=86.18, F1-score=85.91	√	Compared with LSTM-CRF, Mention hypergraph, Span-based model.	Propose a nested named entity recognition method for traditional Chinese medicine records.	This paper use word-character-level embedding to enable the model to better learn semantic and syntactic information, two-layer labeling strategy allows the model to better extract fine-grained results. This work provides a good nested NER method for TCM records, which can extract valuable knowledge from previous TCM books and records	Entity boundary problems caused by the difference between words and characters in TCM corpus, many methods that have great performance on the English datasets are not suitable for the NER task in TCM field
49	Guan Y et al., 2021	A Traditional Chinese Medicine Terminology Recognition Model Based on Deep Learning: A TCM Terminology Recognition Model	2021	CA	Ancient literature	TCM prescription in 517 ancient TCM books	Not specified	None	BERT-BiLSTM-CRF	Deep learning	Supervised	Disease, pattern, symptom	Entity extraction	Precision=86.54, Recall=88.59, F1-score=87.55, when the size of training set is 10000	√	Compared with BiLSTM(word2vec), BiLSTM-CRF(word2vec), BERT-BiLSTM	Presented a traditional Chinese medicine terminology recognition model named BERT-BiLSTM-CRF	Transferring a BERT model trained on other large-scale Chinese corpora as the pre-training model benefits the TCM terminology recognition, and CRF plays an important role in modeling the contextual label features of sequences in TCM term recognition tasks.	(1) there are some characteristics in Chinese text, such as fuzzy word boundary and lack of explicit morphological features of entities, which restrict the performance of NER model for Chinese text; (2) how to apply the most advanced deep learning model to the task of TCM term recognition.
